# Host specificity and adaptive evolution in settlement behaviour of coral-associated barnacle larvae (Cirripedia: Pyrgomatidae)

**DOI:** 10.1038/s41598-023-33738-3

**Published:** 2023-06-14

**Authors:** Fook-Choy Yap, Hsi-Nien Chen, Benny K. K. Chan

**Affiliations:** 1 Biodiversity Research Center, Academia Sinica, Taipei, 11529 Taiwan; 2 Chemistry and Environmental Research Laboratory, Taiwan Power Research Institute, New Taipei City, 238 Taiwan; 3 Present Address: Graduate School, University of Nottingham Malaysia, Jalan Broga, Selangor 43500 Semenyih, Malaysia

**Keywords:** Behavioural ecology, Biodiversity, Biooceanography, Conservation biology

## Abstract

Coral-associated organisms often exhibit a continuum of host specificities. We do not know whether the variation in host specificity is related to the settlement organs or preferential settlement behaviours of the larvae. We examined the morphology of attachment discs, the settlement and metamorphosis of coral barnacles—*Pyrgoma cancellatum* (lives in a single coral species), *Nobia grandis* (two families of corals), and *Armatobalanus allium* (six families of corals). Our results revealed that the attachment organ of all three species are spear-shaped with sparse villi, indicating that the morphology of the attachment organs does not vary among species with different host specificities. Larvae of *P. cancellatum* and *N. grandis* only settle on their specific hosts, suggesting that chemical cues are involved in the settlement. Cyprids of *N. grandis* display close searching behaviour before settlement. Cyprids of *P. cancellatum* settle immediately on their specific host corals, without any exploratory behaviour. The host specificity and exploratory behaviours of coral barnacle cyprids are results of adaptive evolution. We argue that there is a trade-off between exploration and energy conservation for metamorphosis processes. Coral barnacle metamorphosis is longer when compared to free-living species, likely because it involves the development of a tube-shaped base on the coral surface.

## Introduction

Coral reefs support a high diversity of fauna, of which more than 56% form obligate symbiotic relationships with their host corals^1–3^. Coral-associated faunas include generalists that can live in a wide range of corals and specialists that live in a narrower range of hosts. Specialisation to host corals promotes the morphological evolution and speciation in symbiotic species, contributing to a considerable portion of the marine biodiversity^2,4–7^. Inferring how the symbiotic species select and adapt to their host corals is essential to elucidate the processes that generate and determine the observed distribution of reef fauna diversity.

Symbiotic fauna starts their life with their hosts during the settlement stage as planktonic larvae^8–13^. Larvae of generalists can settle on a wide variety of hosts. Larvae of specialists may also settle on a variety of hosts, but those settled on inappropriate hosts eventually suffer from post-settlement mortality. Alternatively, specialist larvae can actively select their preferred hosts for settlement. Only a limited number of studies have investigated this hypothesis because it is difficult to culture the larvae of coral-associated invertebrates and maintain coral health under laboratory conditions. A recent study on sponge barnacle larvae revealed that the settlement organs of cyprids are specialised to different sponge host types, suggesting that larvae can preferentially settle on their specific hosts^14^.

**Figure 1:**
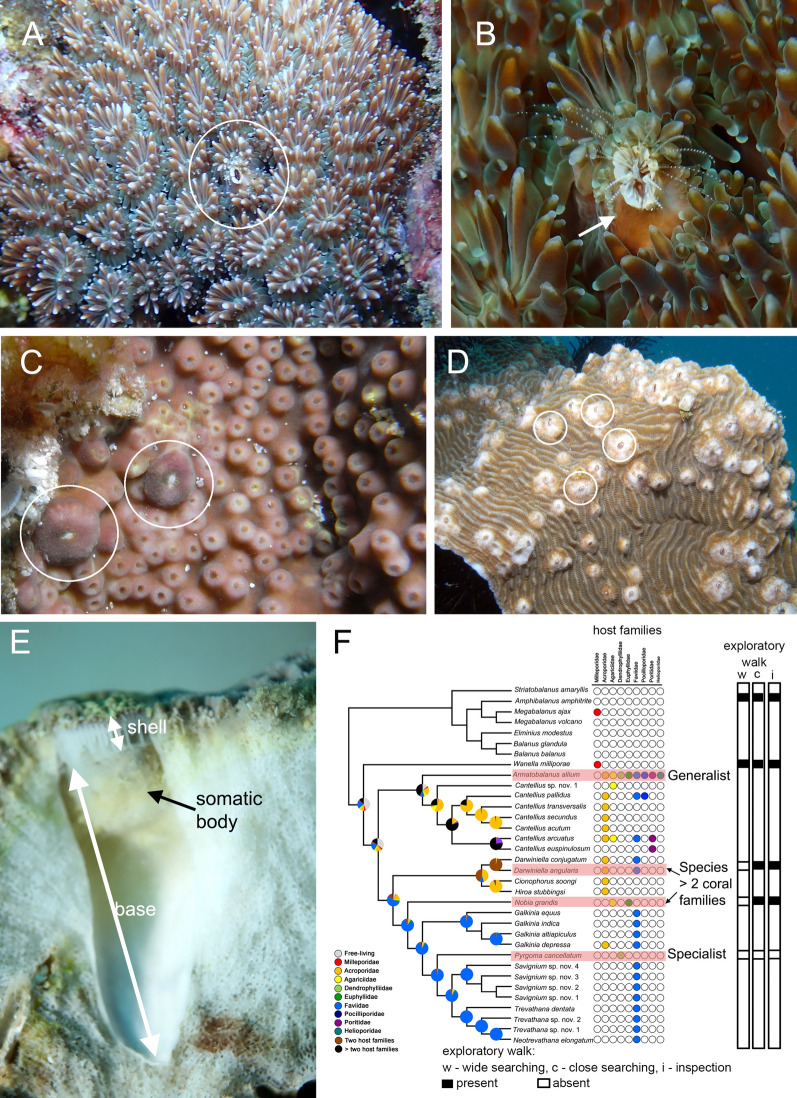
(**A**) Adult coral-associated barnacle *Nobia grandis* (white circle) inhabiting a host coral, a species of *Galaxea*. (**B**) Enlarged view of *Nobia grandis*. (**C**) A specialist species, *Pyrgoma cancellatum* living on a host coral, a species of *Turbinaria*. (**D**) *Armatobalanus allium* living on the host coral *Pachyseris*. (**E**) Longitudinal section of corals bearing coral barnacles showing the cup-shaped base, double arrow in base indicate the vertical base length. (**F**) Phylogenetic tree of coral-associated barnacles with host usage patterns adapted from Tsang et al.^22^ and the selected species (highlighted in pink) in the present study, covering a range of host specificities. Exploratory behaviours in *A. amphitrite*, *W. milleporae* and *D. angularis* were extracted from Lagersson and Høeg^23^, Yap et al.^24^ and Liu et al.^25^, w - walking, c - close searching, i - inspection.

Coral-associated barnacles (Cirripedia: Sessilia: Pyrgomatidae) are a common group of obligate symbionts of scleractinian corals^15–17^ (Fig. 1A–D). Their external calcified shell is located at the same level as the coral surface, and the coral tissue often grows over the barnacle (Fig. S1). The base of coral barnacles is cup-shaped and embedded in the skeleton of the host coral (Fig. 1E). Hiro^18^ observed that once a barnacle starts to grow on a coral after settlement and metamorphosis, it can expand its shell width until the base forms on the skeleton. Further increases in width are then slowed down, but the upward growth keeps in pace with that of the corals. This suggests that coral barnacles can modify their base as the coral grows. However, how the base of the coral-associated barnacle forms is still unknown. Pyrgomatid coral barnacles (except *Hoekia*) are primarily suspension feeders, but ^13^C stable isotope studies showed that the organic matter produced by the coral zooxanthellae is one of the carbon sources for the resident barnacles^19,20^. In return, the ammonium released from the coral barnacles is absorbed by the coral’s zooxanthellae^20^. The symbiotic relationship between the barnacles and the corals is considered to be mutualistic. To date, there are 93 species of coral barnacles worldwide, with variable degrees of host specificity^21^.

Molecular phylogenetic studies revealed that the pyrgomatid barnacles are a monophyletic group, being sister to the clade of the fire coral barnacle Wanellinae^21^, and the clades of Wanellinid and Pyrgomatid barnacles are sister to the free living Balanid species^22^. Within the Pyrgomatinae, barnacles are composed of two sub-clades: the *Cantellius* clade and the major clade (which are comprised entirely of genera of Pyrgomatinid coral barnacles^22,26^). There is a phylogenetic trend in the degree of host specificity of coral barnacle species (Fig. 1F). The generalist species (*Armatobalanus*, which can live in eight different coral families) is located at a sister position to the *Cantellius* clade, which consists of generalists and specialists^22^. The relatively specialist species (e.g., species of *Trevathana* and *Savignium*, some of which can live in only a few hosts or from a single coral family) are positioned at the major clade^22^ (Fig. 1), being sister to the *Armatobalanus* + *Cantellius* clade.

The life cycle of barnacles consists of the sessile adult and plankton larval phases^27^. Pyrgomatid coral-associated barnacles have six naupliar and one final cypris stages^25,27^. Cyprids use a pair of antennules to walk in a bipedal manner on the substratum to search for suitable habitats for settlement^28^. Cyprids antennules are composed of four segments^28,29^. The third segment is the attachment organ, also called attachment disc, which has dense villi^30^. The fourth segment has an array of terminal and subterminal setae, believed to detect the physical and chemical properties of the substratum (Fig. S1). The variation in the shapes of attachment discs among free-living and specialised barnacles that are symbiotic with corals, sponges and fire corals appears to be a result of adaptive radiations. The attachment disc of free living species are bell-shaped and with dense villi. Sponge barnacle cyprids have hook-shaped and shoe-shaped attachment discs that are specialised to different sponge hosts^14^. The larval attachment organs of many coral-associated barnacles were observed to be spear-shaped to allow the organisms to penetrate into the coral tissue for settlement^25,28^.

In free-living barnacles, prior to settlement, cyprids display wide searching (larger steps and higher walking speed), close searching (smaller steps and lower speed) and inspection behaviours (walking ceased, with both antennules attached to a specific spot and moved in a jerky manner)^23^. Presently, settlement and metamorphosis of pyrgomatid coral barnacles have only been recorded in *Darwiniella* (host records include two coral families)^25^. Cyprids of *Darwiniella* perform a close-searching-type exploratory walk on the coral tissue (without the wide searching behaviour in free-living species) to ascertain the correct hosts before settlement. Settlement and metamorphosis inside the coral tissue take about 10 days to complete, longer than those of free-living species (1–2 days).

Larval morphology and settlement have never been examined in specialist coral barnacle species. It is still unknown whether larvae of specialist coral barnacles can settle in a wide range of hosts (those settled on non-preferred hosts suffer from post-settlement mortality) or exclusively on their specific host. If the larvae of specialist species can accurately detect and locate their hosts before settling, then the shape of the attachment organ may differ from that of the generalist species, which can explore and settle on wider ranges of hosts. Do specialists perform any exploratory walks on their hosts, if they exclusively select their hosts before settlement? The present study tests the hypothesis that the attachment organs of specialists are different from those of generalists, and larvae of specialists only settle on their specific hosts and do not perform any exploratory behaviour. Compared to the exploratory behaviour from free-living barnacles (wide searching, close searching and inspection), exclusive close searching or lack of searching behaviour during settlement in coral barnacles is a result of adaptive evolution.

## Results

**Figure 2:**
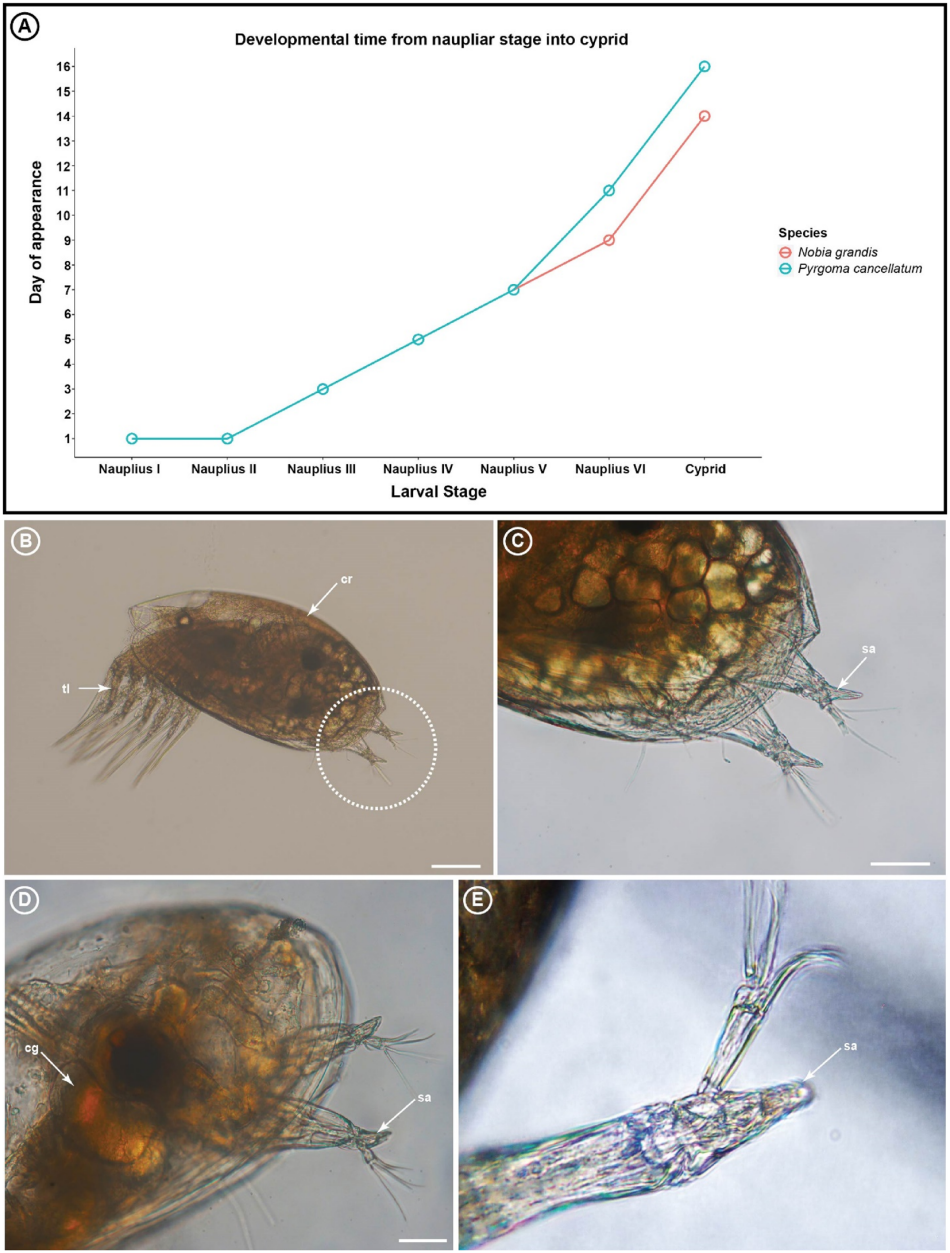
(**A**) Time taken for *Nobia grandis* and *Pyrgoma cancellatum* to develop from the naupliar stage into the cyprid stage*.* (**B**) The cyprid of *N. grandis* possesses a pair of highly specialised spear-shaped attachment organs (dotted circle). (**C**) Enlarged view of the spear-shaped attachment organs (sa) of *N. grandis*. (**D**) Spear-shaped attachment organs of *P. cancellatum*. (**E**) Enlarged view of attachment organs of *Armatobalanus allium.* Scale bars: 100 µm (b), 50 µm (c, d).

Larvae of the coral-associated barnacles *Armatobalanus allium*, *Nobia grandis,* and *Pyrgoma cancellatum* were chosen as the model organisms because they portray different degrees of host specificity. *Armatobalanus allium* has the lowest degree of host specificity, living in six different families of host corals. *Nobia grandis* has a medium degree of host specificity as it is found inhabiting two different families of corals—Euphylliidae and Agariciidae. *Pyrgoma cancellatum* is an extreme specialist, found only on *Turbinaria* corals. Larvae of *A. allium* were collected from wild plankton samples in the Northeast coast of Taiwan. The species identity of *A. allium* larvae was confirmed by DNA barcoding using the COI gene in Chen et al.^31^. Larvae of *N. grandis* and *P. cancellatum* were obtained from laboratory cultures. Both *Nobia* and *Pyrgoma* took 14–16 days to complete their larval life cycles (from naupliar to cyprids; Fig. 2A). Comparison of morphology of attachment organs were based in *A. allium*, *N. grandis* and *P. cancellatum*. Settlement experiment and settlement and metamorphosis observations were only conducted in *N. grandis* and *P. cancellatum*.

## (a) Morphology of cyprid attachment organ in *Armatobalanus allium*, *Nobia grandis,* and *Pyrgoma cancellatum*

**Figure 3:**
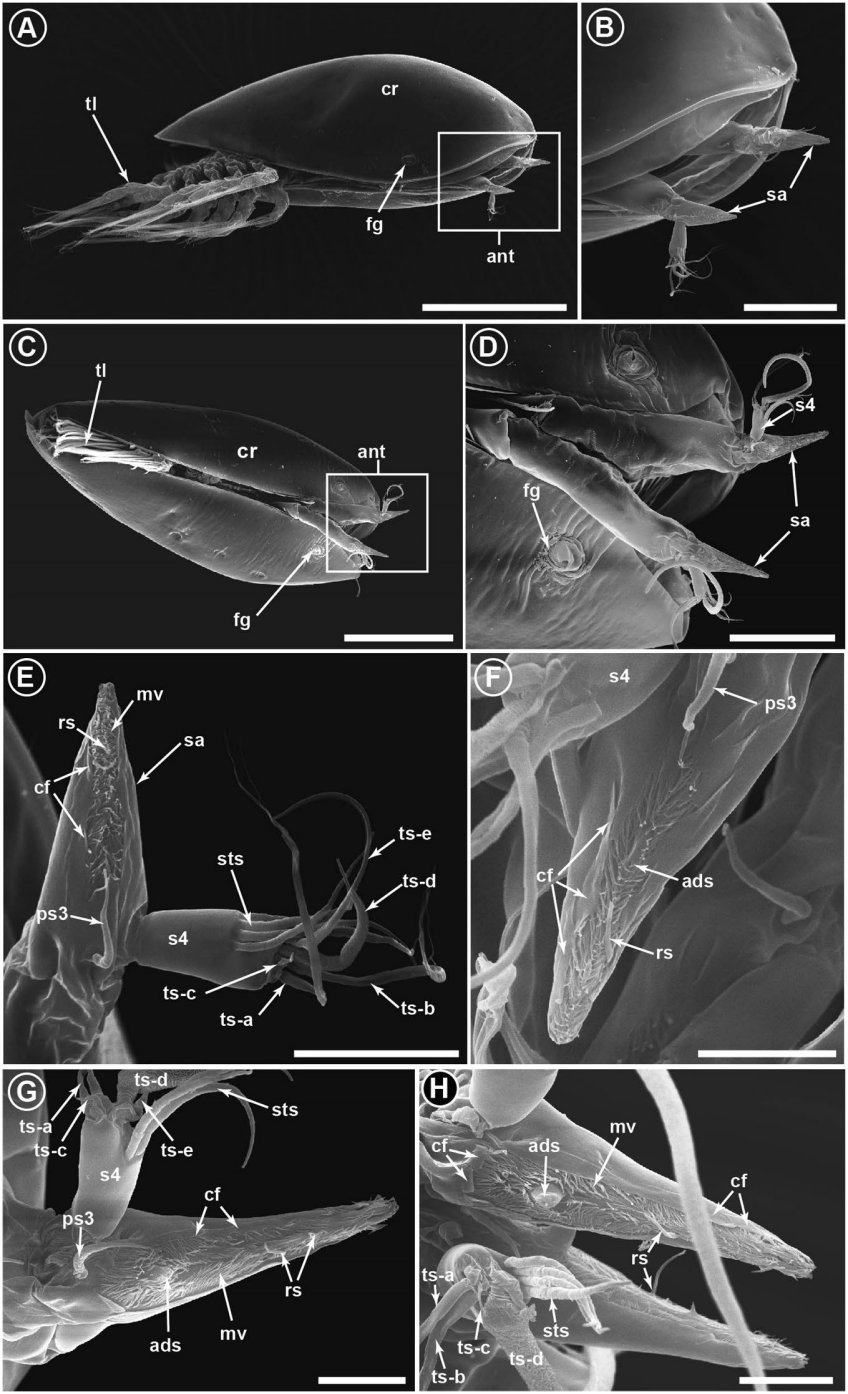
Scanning electron microscope images of the cyprids of *Nobia grandis* (**A**,**B**,**E**,**F**) and *Pyrgoma cancellatum* (**C**,**D**,**G**,**H**) and their attachment organs. (**A**) The whole cyprid of *N. grandis* with antennules (ant) extended (square box). (**B**) The spear-shaped attachment organs (sa) of *N. grandis*. (**C**) Overview of the *P. cancellatum* cyprid with antennules extended (square box). (**D**) A spear-shaped attachment organ located in the third antennular segment of *P. cancellatum*. (**E**) Enlarged view of the spear-shaped attachment organs of *N. grandis*. The attachment disc is surrounded by microcuticular villi (mv). The fourth antennular segment (s4) carries five terminal setae (ts-a to ts-e) and four subterminal setae (sts). (**F**) The attachment disc is encircled by a series of cuticular flaps (cf). An axial disc seta (ads) is located in the proximal region of the attachment disc. (**G**) Enlarged view of the spear-shaped attachment organs of *P. cancellatum*. The attachment disc is covered with the microcuticular villi. Radial setae (rs) are located at the periphery and the tip of the attachment organ. (**H**) Cuticular flaps of irregular sizes and shapes surround the attachment disc of *P. cancellatum*. cr, cyprid carapace; fg, frontolateral gland pore; ps3, post-axial seta 3; tl, thoracic limbs. Scale bars: 200 µm (a), 150 µm (c), 50 µm (b, d), 20 µm (f), 10 µm (e, g, h).

Light microscopy revealed that the attachment organs of *A. allium*, *N. grandis,* and *P. cancellatum* were spear-shaped with a sharp-pointed attachment organ (Fig. 2B–E). Based on the SEM photomicrographs, the third antennular segments of *N. grandis* and *P. cancellatum* appeared to taper distally until it reached the end of the tip, forming a spear-shaped attachment structure (Fig. 3A–D). The ventral surface of the third antennular segment possessed a narrow attachment disc enclosed by microcuticular villi (Fig. 3E–H). The attachment disc was surrounded by irregular-size and -shaped cuticular flaps (Fig. 3E–H). An axial disc seta extended from the proximal region of the attachment disc via an opening pore (Fig. 3F–H). Four radial setae were found extending from the periphery of the attachment disc and located near the tip of the attachment organs (Fig. 3F–H). Two spine-like cuticular structures were found near the distal end of the attachment disc. Postaxial seta 3 was located proximally to the attachment disc and at the same level as the articulation of the fourth antennular segment (Fig. 3E,G). Similar to other barnacle cyprids, the fourth segment carried five terminal setae (A–E) and subterminal setae (Fig. 3E,G,H).

## (b) Settlement of *Nobia grandis* and *Pyrgoma cancellatum* in different host corals

Experimental results revealed that the coral barnacle host specificity significantly affected the cyprid settlement of both coral-associated barnacles. The cyprids of *N. grandis* and *P. cancellatum* were only found settling (60% successful settlement) on their respective host corals, *Galaxea* sp. and *Turbinaria* sp. (all the *Galaxea* and *Turbinaria* used for experiments were collected from the respective same colonies to ascertain they belong to the same species). When exposed to non-host corals, the cyprids were observed to swim away from the corals upon exposure. Moreover, some cyprids became inactive and remained at the bottom of the water column after several hours of exposure to the non-host corals.

## (c) Settlement behaviours and metamorphosis of *Nobia grandis* and *Pyrgoma cancellatum*

Observations under the stereomicroscope showed that the cyprids of *N. grandis* swam towards the host coral upon exposure and landed on the coral surface with their highly specialised antennules. Once the cyprid stabilised its position, it began to walk on the epidermal layer of the host coral, indicating the start of an exploratory event. The cyprid exploratory event of *N. grandis* consisted of two behaviours: close searching and inspection. The exploratory event of *N. grandis* ceased when the cyprid permanently settled onto the calcareous skeleton of the host coral. Similar to *N. grandis*, the cyprid of *P. cancellatum* swam directly towards its host coral, a species of *Turbinaria*, upon exposure and subsequently landed on the coral surface by immediately penetrating its sharp antennules into the coral’s epidermal layer. There was no walking behaviour during the cyprid exploratory event of *P. cancellatum* after the cyprid attachment organ penetrated the coral tissue. The settlement location of *P. cancellatum* includes all positions of the coral polyps including tentacles and corallite position.

### Close searching

Close searching behaviour was only observed in *Nobia grandis* but not *Pyrgoma cancellatum* (no searching behaviour). Cyprids of *N. grandis* engaged in a slow walking movement during the close searching event. Based on the video analysis, the cyprid used its attachment organ to penetrate the epidermal layer of the coral while walking on the coral surface (Movie 1). The cyprid took an average of 24 s (14–42 s, n = 17) for each antennular step in the walking movement. The probing activity of the cyprid attachment organ was not noted as it penetrated the coral’s epidermal layer. When encountering an unsuitable settlement area, the cyprid retracted both its antennules and swam away from the host coral.

The walking movement of the cyprid was like the motion of an inverted pendulum swing (Movie 1). During the movement, the cyprid retracted one of the penetrated antennules into the mantle cavity, while keeping the other penetrated in the host coral (Movie 1). In this period, the cyprid body was elevated and remained parallel to the surface of the host coral. The cyprid subsequently extended the retracted antennular from the mantle cavity to took a short antennular step, followed by the slow penetration of the attachment organ into a new spot on the coral’s epidermal layer (Movie 1). Occasionally, the coral tissue would be dragged by the walking movement of the cyprid (Movie 1). We observed that the cyprid initiated a directional change when encountering an unfavourable settlement area or difficulty retracting its antennules.

### Inspection

#### Nobia grandis

The walking behaviour of the *Nobia grandis* cyprids terminated upon the inspection event. At this point, the cyprid was in a static position on the potential settlement area (Movie 2). Observation under the stereomicroscope revealed that the second and third antennular segments of attachment organ were embedded into the epidermal layer of the coral. This indicated that the antennules were continuously retracted and extended to penetrate deeper into the epidermal layer until the attachment organ touched the calcareous skeleton of the coral. The thoracic limbs were observed to beat occasionally during the inspection behaviour. The sudden beatings of the thoracic limbs caused a jerky movement in the cyprid body.

#### Pyrgoma cancellatum

The cyprids of *P. cancellatum* penetrated its attachment organ into the coral tissue upon landing on the coral (Movie 3). This penetration immediately triggered the release of mucus by the host coral. The cyprid body was eventually covered with this mucus and surrounded by the coral’s digestive mesenterial filaments.

### Settlement

#### Nobia grandis

The antennular movement and intermittent beatings of thoracic limbs ceased during the settlement phase. The second and third antennular segments remained embedded in the epidermal layer of the coral (Movie 2). The anteroventral region of the cyprid body rested on the coral surface at ~ 45°. The actual secretion of permanent adhesives was not noted, but momentary contractions of the muscular sac and cement glands were found to occur near the cyprid compound eyes (Movie 2). This suggests that the permanent adhesives from the cement glands may have secreted onto the calcareous skeleton of the coral at this point (Movie 2). Occasionally, the permanently settled cyprid would be surrounded by digestive mesenterial filaments of the host coral. Examination under the stereomicroscope showed that the cyprids of *N. grandis* settled randomly on the host coral, with two individuals settling near the apical region of the coral polyps, five on the lateral of coral polyps, and one on the coral’s surface between the coral polyps.

#### Pyrgoma cancellatum

After several minutes of exposure, the second and third antennular segments of attachment organ penetrated the epidermal layer of the coral, indicating the start of the cyprid’s inspection behaviour (Movie 3). During this phase, the anterior region of the cyprid body rested on the coral surface. The cyprid’s thoracic limbs were beating sporadically.

### Metamorphosis

#### Nobia grandis

**Figure 4:**
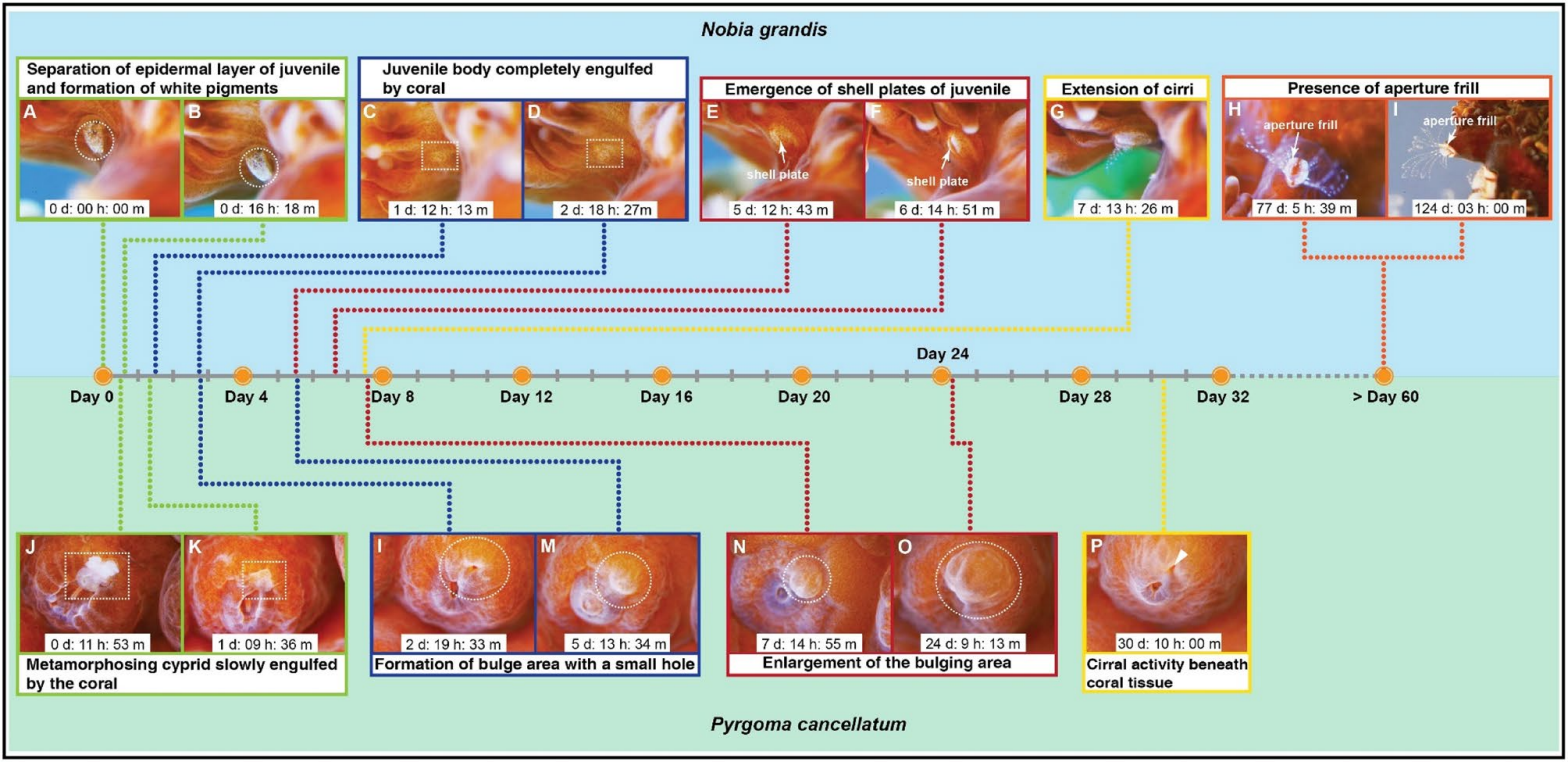
Metamorphosis processes of *Nobia grandis* (**A**–**I**) and *Pyrgoma cancellatum* (**J**–**P**) after the cyprids permanently settled on their respective host corals.

The metamorphosis from cyprid to juvenile occurred immediately after the cyprid secured its permanent attachment on the host coral. The cyprid took about 7 days to complete the transition into juvenile, from permanent settlement to the extension of juvenile cirri. During the early metamorphosis process, the epidermal layer of the juvenile separated from the cuticle of the cyprid (Fig. 4A; Movie 4). White pigments were also observed around the anterior of the cyprid body at this point (Fig. 4A; Movie 4). The white pigments became more prominent as the metamorphosis proceeded (Fig. 4B; Movie 4).

About 36 h later, the shed carapace was found in the water column, indicating the completion of the transitional stage of cyprid into juvenile. A slight bulge area on the coral surface was observed on the settlement spot of *N. grandis* (Fig. 4C; Movie 4). The juvenile barnacle appeared to be entirely engulfed by the host coral and remained inactive (Fig. 4C; Movie 4). This showed that the newly developed juvenile body may have slipped out from the cyprid carapace and attached to the coral’s calcareous skeleton. The bulge area on the coral surface expanded as the metamorphosis proceeded (Fig. 4D-F; Movie 4). This suggested that the growth of juvenile barnacles may have continued beneath the coral tissue.

After 5 days, shell plates of the juvenile were observed on the bulge area of the coral surface (Fig. 4E, F; Movie 4). This likely resulted from the growth of the juvenile's base, causing the shell plates to be pushed against the epidermal layer of the coral tissue (Fig. 4E, F; Movie 4). The juvenile feeding cirri were extended beneath the coral tissue. The continuous push of the shell plates and extension of the cirri eventually slit the epidermal layer of coral, which led to the emergence of the shell plates on the coral surface (Fig. 4F; Movie 4). About 24 h later, the shell plates of the juvenile emerged on the coral surface with active cirral activity (Fig. 4G; Movie 4). The aperture frill of the barnacle was not observed during this period, but it developed as the juvenile grew (Fig. 4H, I; Movie 4).

**Figure 5:**
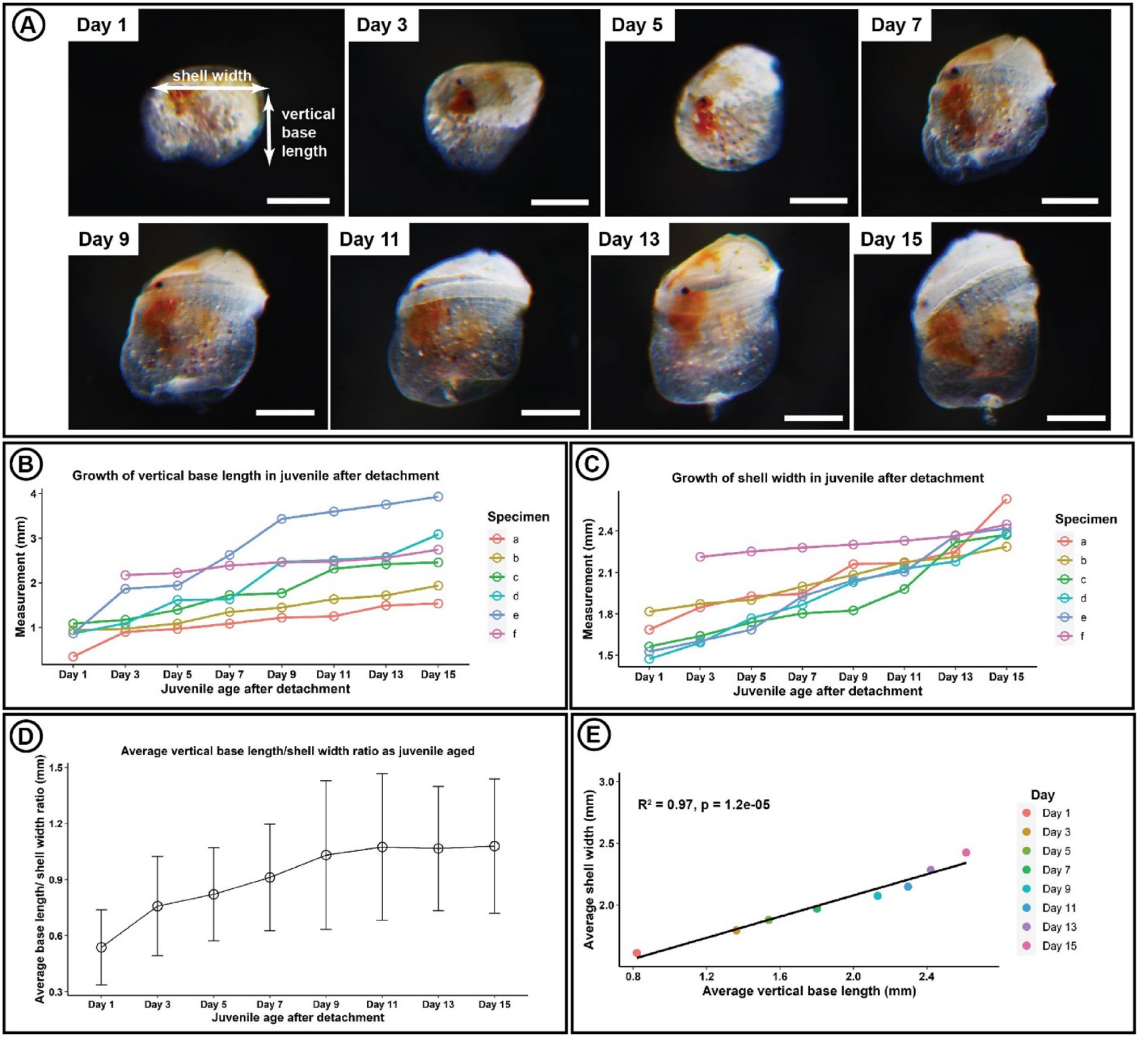
Growth pattern of the juvenile’s base of *Nobia grandis*. (**A**) Different age groups of *N. grandis* juvenile. (**B**) Growth of vertical base length in the juvenile of *N. grandis*. (**C**) Growth of shell width in the juvenile of *N. grandis*. Note: One-day-old juveniles of *N. grandis* (a to e) were collected after shedding the cyprid carapace. A three-day-old juvenile of *N. grandis* (f) was collected after the host coral began to bleach. (**D**) Average growth of vertical base length/shell width ratio of *N. grandis.* (**E**) Linear regression of average vertical base length and shell width in the juveniles of *N. grandis.* Note the growth of the base and shell of *N. grandis* can be observed after the barnacle was removed from host corals using fine needles. The juvenile age after detachment in the X-axis of B-D refer the age after the barnacles were removed from the hosts.

The base of the *N. grandis* juvenile appeared almost transparent at the early stage, and slowly mineralised as it grew (Fig. 5A). Remnants of the cypris eyespots can be seen at the base of shell (Fig. 5). Experimental results showed that the vertical base length and shell width increased as the juvenile aged (Fig. 5A–D). The growth in the vertical base length and shell width varied among the juveniles of *N. grandis* (Fig. 5B, C). The average vertical base length and shell width ratio of *N. grandis* consistently increased from Day 1 to 11 (Fig. 5D), indicating that the base length developed more rapidly than the shell width. Figure 5 shows a positive correlation between the average growth of the vertical base length and shell width of *N. grandis* (R^2^ = 0.97, *p* < 0.001, Fig. 5E).

#### Pyrgoma cancellatum

The permanent settlement of the cyprid onto the host coral occurred within 6 to 8 h of the inspection event. The settled cyprid was dragged into the coral when the digestive mesenterial filaments retracted (Fig. 4J; Movie 5). The shed cyprid carapace was found in the water column, indicating that the cyprid’s metamorphosis may have occurred when the coral was dragging the cyprid. However, the metamorphic event of the cyprid was not observed as the digestive mesenterial filaments of coral surrounded the cyprid. It took about 24 h for the host coral to engulf the cyprid (Fig. 4K; Movie 5). Once the engulfment process was complete, a small bulging area with a hole on the surface was found in the settlement area of *P. cancellatum* (Fig. 4L; Movie 5). The metamorphosed juvenile barnacle was located beneath this bulging area on the coral and remained inactive (Fig. 4M). The bulging area became more prominent (Fig. 4M–O; Movie 5), likely because of the growth of the juvenile beneath the coral tissue. The cirri were extended beneath the coral surface, and this was observed through the expanded hole on the surface of the bulging area (Fig. 4P; Movie 5).

## Discussion

Barnacle lifestyles cover a wide range of habitats, including free living in the intertidal zone and deep sea, epibiotic on cetaceans and sea turtles, and living with corals and sponges^15,16,32–37^. Previous studies found that the attachment disc of coral-associated barnacles is spear-shaped, which allows these barnacles to thrive on specific host corals^25,28^. The coral-associated arcothoracican barnacle *Berndtia* and the thoracican pyrgomatid barnacles are two distantly related barnacle clades (superorder level) in the molecular phylogenetic tree and from ancestors with fundamentally different antennular phenotypes, these two clades of barnacle have spear-shaped antennules suggested spear shaped antennules are evolved independently for settling in scleractinian corals. The present study detailed the morphology of attachment organs, settlement, and metamorphosis of coral barnacles with different host specificities. We found that the attachment organs of the *Armatobalanus allium* (low degree of host specificity), *Nobia grandis* (living in two families of corals), and *Pyrgoma cancellatum* (high degree of specificity) are allspear-shaped, suggesting that the shape of the attachment organ does not relate to the coral’s host specificity.

The attachment organs of barnacle cyprids are often equipped with dense microcuticular villi. Until now, the function of the microcuticular villi was under debate, with most studies suggesting that they increase the contact surface area for attachment, but none offering definitive proof of this^38–41^. Cyprids of species on high-energy rocky shores appear to have a higher density of villi on the attachment disc than the deep-sea or epibiotic species^30^. Variation in the distributions of microcuticular villi on the attachment organs of sponge-inhabiting barnacles has been proposed to be a morphological adaptation of barnacles to respond to the surface characteristic of the hosts^14^. In coral barnacles, the attachment organ is spear-shaped and the villi region is much reduced, with sparse villi observed. Despite the differences in host specificity, the villi distribution and density of *N. grandis* and *P. cancellatum* are similar to other coral-associated barnacles, such as *Darwiniella*^25^, *Savignium,* and *Trevathana*^28,35^. Therefore, this study suggests that the host specificity of the coral-associated barnacles does not rely on the external morphological adaptation of the attachment disc, but instead is likely related to the chemical cues between the barnacles and host corals. The spear-shaped attachment disc in coral-associated barnacles is essential for settlement on scleractinian corals and is a result of adaptive evolution^42^.

Based on larval biology studies of the model barnacle species *Amphibalanus amphitrite* (a free-living intertidal species), the inducing of cyprid settlement has always been linked to the settlement-inducing protein complex (SIPC), a chemical cue released by the conspecific adult barnacles that settle on non-living surfaces^43–47^. The SIPC hypothesis does not seem to explain the settlement of coral-associated barnacles since there are distinct differences in the substratum preferences and settlement strategies. Our study revealed that the settlement responses of coral barnacle cyprids are mainly affected by their respective host coral. By exposing *N. grandis* to various coral species, it was observed that its exploratory behaviours were only triggered by the presence of its specific host coral. Intriguingly, the negative responses from *N. grandis* occurred when it was exposed to non-specific host corals, causing the inhibition of the exploratory behaviours, and the cyprids tended to swim away from the corals. Similar settlement responses are also noted in another coral-associated barnacle: *Darwiniella angularis*^25^. Taken together, these observations suggest that the occurrence of chemical communication between the barnacles and their respective host corals is a prerequisite for coral-associated barnacle cyprids to begin settlement behaviours. A similar settlement behaviour was observed in the sponge-inhabiting barnacles, including *Balanus trigonus* and Acastine barnacles, which settle on sponges with the presence of chemical cues, including the metabolites^14,48^.

Our study revealed that coral barnacles with different degrees of host specificity display different exploratory behaviours when encountering their respective host corals. Barnacle cyprid’s exploratory behaviours involve a sequence of events—consisting of wide searching, close searching, and inspection—before the permanent settlement onto a suitable substratum or habitat^23,49,50^. However, some of these events were absent in the exploratory behaviours of *N. grandis* and *P. cancellatum*. After exposure to their host corals, cyprids of *N. grandis* immediately engaged in a close searching behaviour, whereas the cyprid of *P. cancellatum* began inspecting its host coral upon exposure and did not carry out any searching events. One possible explanation for these scenarios is that searching events represent a trade-off, as they require energy. We found that both coral barnacles struggled with their walking activities during the exploratory behaviours. Any struggle that a cyprid has during exploration depletes its energy reserve, which may disrupt the settlement and metamorphosis processes. Recent studies quantifying barnacle settlement behaviours also observed a possible trade-off in the exploratory behaviour of barnacles, which reveals a significant decline in walking activity and an increase in settlement rate in older cyprids^51^. This shows that older cyprids probably reduce their walking activity during the exploratory phase to conserve energy and ensure that they permanently settle onto the substratum^50^. Moreover, coral barnacles have a longer metamorphosis process than other free-living barnacle species^25^, indicating that the cyprid may have to use more energy to complete metamorphosis (Table 1). Therefore, the trade-off is necessary for energy conservation in cyprids to ensure that they settle permanently and complete metamorphosis. These differences in the exploratory behaviours and the time length of metamorphosis between coral-associated barnacles and free-living species appear to be the result of adaptive evolution events (Fig. 1F).

**Table 1:** Summary table on the exploratory behaviours, settlement and metamorphosis of pyrgomatid barnacles and the fire coral associated barnacle *Wanella milleporae*.

Barnacle species	*Darwiniella angularis*	*Nobia grandis*	*Pyrgoma cancellatum*	*Wanella millepora*
Host coral usage	Faviidae and Acroporidae	Euphylliidae and Agariciidae	Exclusive in *Turbinaria* corals	Exclusive in fire corals *Millepora*
Exploratory behaviour	Close searching and inspection (without wide searching)	Close searching and inspection (without wide searching)	Without any exploratory behaviour, only inspection prior to settlement	Wide searching, close searching and inspection
Shape of attachment organ	Spear-shaped	Spear-shaped	Spear-shaped	Bell-shaped
Larval development period	14 days	14 days	16 days	11 days
Settlement process	Penetrate into coral tissue	Penetrate into coral tissue	Penetrate into coral tissue	Settle on fire coral surface
Metamorphosis period	10 days	7 days	30 days	1–2 days
Literature	Liu et al.^25^	Present study	Present study	Yap et al.^24^

The walking behaviour during the cyprid’s exploratory phase appears to be invasive to the host coral, as the antennule is required to penetrate the tissue of the host coral^25,28^. This action of antennular penetration punctures numerous holes on the coral tissue surface, which results in the coral releasing digestive filaments. Previous studies reported that the release of digestive filaments is a defensive response against the settling cyprids^25^. The authors also revealed that the settling cyprids are capable of enduring attacks from the digestive filaments by penetrating further into the coral tissue. In the present study, some settling cyprids were surrounded by the digestive filaments during settlement and metamorphosis. Intriguingly, this did not have an immobilisation effect on the cyprids, even though they were surrounded tightly by the digestive filaments, which are packed with nematocysts. Moreover, the retraction of digestive filaments appeared to have contributed to the settlement of coral barnacles by pulling the cyprid into the coral tissue, allowing the cyprids to settle onto the coral endoskeleton. All these behavioural observations suggest that the release of digestive filaments by the coral may be a reactional response to the antennular penetration instead of a defensive response. Similar observations were reported in the fire-coral-associated barnacle *Wanella milleporae*, in which cyprids of *Wanella* can tolerate, attack and deactivate the fire coral polyps during the walking behaviour prior to settlement. After settlement, calluses will be formed by the fire corals to enhance the survival of the settlers^24^.

Something noteworthy is that it appears to take much longer for a permanently settled cyprid to metamorphose into a juvenile with an active cirrus in coral-associated barnacles. In this study, the coral barnacles *N. grandis* and *P. cancellatum* took about 7 and 30 days, respectively, to complete their metamorphosis processes. This long metamorphosis time has also been noted in the coral barnacle *Darwiniella angularis*^25^, for which the authors suggested that the metamorphosis of coral barnacles is prolonged due to the time it takes for the barnacle to tolerate attacks from the digestive filaments of corals during settlement. However, our results suggest an alternative hypothesis that the reason metamorphosis takes longer in coral barnacles is due to the development of the tube-shaped base. As observed in *N. grandis*, the newly metamorphosed juvenile had a short base, which was presumably attached to the coral exoskeleton when engulfed by the host coral. This short base of *N. grandis* grew significantly as the juvenile continued to age (Fig. 5). This growth of the juvenile's base is suggested to be an important event for the emergence of the shell plates of barnacles on the coral surface. Thus, the failure of the juvenile to develop its base may be a major threat to the coral barnacles.

Post-settlement growth of coral-associated barnacles has been proposed to focus mainly on the shell width in the early stage, while the development of vertical base length occurs much later^18,24^. The vertical growth of base length has also been suggested to be the barnacle's response to prevent being overgrown by the host corals^18,31^. However, our study seems to contradict these two hypotheses, as the base depth (known as vertical base length in this study) and shell width of the newly metamorphosed juvenile were found to develop simultaneously (Fig. 5). Moreover, the vertical base length of *N. grandis* appeared to grow more rapidly than the shell width (Fig. 5). This study also revealed that the base of *N. grandis* can grow vertically without the presence of its host coral. Therefore, we suggest that the lifetime vertical growth of the base length may be a natural growth pattern of coral-associated barnacles instead of an outcome influenced by the growth of host corals.

In general, our results revealed that coral barnacle generalists and specialists share the same antennular morphology without further adaptations to the spear-shaped attachment organ, despite inhabiting a variety of scleractinian corals. This indicates the mechanical function needed to settle on a host coral is similar across the Scleractinia. The specific adaptation that determines host specialization in coral barnacles may be controlled by behavioural or chemical cues that allow the cyprid to locate and settle on its specific host coral. This study also suggests that the settlement behaviours of coral barnacles could have evolved from adaptive evolution in response to the characteristics of the host corals. Coral barnacles must endure trade-offs to ensure their survival in niche hosts. Future studies on the chemical communications between barnacles and host coral are required to identify the potential metabolites that may stimulate the settlement of coral barnacle and the origin of those metabolites.

## Materials and methods

- Sample collections

Larvae of the coral-associated barnacles *Armatobalanus allium*, *Nobia grandis,* and *Pyrgoma cancellatum* were chosen as the model organisms because they portray different degrees of host specificity. *Armatobalanus allium* has the lowest degree of host specificity, living in six different families of host corals. *Nobia grandis* has a medium degree of host specificity as it is found inhabiting two different families of corals—Euphylliidae and Agariciidae. *Pyrgoma cancellatum* is an extreme specialist, found only on *Turbinaria* corals. Larvae of *A. allium* were collected from wild plankton samples in the Northeast coast of Taiwan. The species identity of *A. allium* larvae was confirmed by DNA barcoding using the COI gene in Chen et al.^52^. To obtain the larvae of *N. grandis* and *P. cancellatum*, the host corals—*Galaxea* and *Turbinaria* species—were collected from Green Island, Taiwan through scuba-diving at 10–20 m deep. Small fragments of host corals (~ 3 cm × 3 cm) with 2–5 associated barnacles were collected using a chisel and hammer. In addition, small fragments of scleractinian coral—such as *Montipora* sp. and the hydrocoral *Millepora tenera—*without any living barnacles were also collected for subsequent settlement experiments. All the collected coral fragments were transported back to the laboratory and maintained in aquarium facilities.

(b) Larval rearing and culturing

The collected coral fragments of *Galaxea* and *Turbinaria* species and their respective associated barnacles *N. grandis* and *P. cancellatum* were kept separately in a 7 L polycarbonate plastic container filled with filtered seawater. The cultures were maintained at 26 °C in a 12:12 h light–dark cycle. The seawater was changed daily. The corals and associated barnacles were fed frozen reef plankton pellets.

The newly released stage I nauplii of *N. grandis* and *P. cancellatum* were collected with the aid of a pointed light source. The nauplii were transferred into sterile Petri dishes (5.5 cm and 9 cm in diameter) containing filtered seawater (filtration: 0.45 µm, 33% salinity). The seawater was changed every two days to avoid waste accumulation. The nauplii were fed mixtures of microalgae (*Isochrysis* sp.*, Chatoceros* sp., and *Skeletonema costatum*) after every seawater change. The larval cultures were kept at 26 °C in a 12:12 h light–dark cycle until they reached the cyprid stage.

(c) Morphology of cyprid attachment organ in specialist and generalist species

Cyprids of *Armatobalanus allium* were only observed under a light microscope as the samples were later used for DNA barcoding for species identification. The cyprids of *N. grandis* and *P. cancellatum* were relaxed in 8% magnesium chloride for 1 h and then washed with 0.45 µm filtered seawater. The morphology of the cyprid attachment organs of these two coral-associated barnacles was photographed using an inverted light microscope equipped with a custom-made C-mount and lens mount adapter attached to a Panasonic Lumix GH4 camera.

To access the detailed structures of the cyprid attachment organs of *N. grandis* and *P. cancellatum*, we used scanning electron microscopy to study the surface morphology of the attachment organs. The cyprids were subjected to the relaxation process as mentioned above and fixed with 2.5 glutaraldehyde (seawater base). The glutaraldehyde fixed cyprids were dehydrated through an ascending series of ethanol, critical point dried, mounted on aluminium stubs, and sputter-coated with gold. The gold coated cyprids were viewed using the FEI Quanta 200 scanning electron microscope operating at 20 kV.

(d) Cyprid settlement on different host corals

To determine the effect of host specificity in the cyprid settlement of coral barnacles, five individuals of three-day-old cyprids of *N. grandis* and *P. cancellatum* were exposed to each of different coral species. For *N. grandis*, the cyprids were introduced to three different scleractinian corals (e.g. *Galaxea* sp. *Turbinaria* sp. and *Montipora* sp.) and one hydrocoral, *Millepora tenera*, while the cyprids of *P. cancellatum* were introduced to *Turbinaria* and *Montipora* corals. *Galaxea* is the host coral of *N. grandis* whilst *Turbinaria*, *Montipora* and *Millepora* are not. *Turbinaria* is the host coral of *P. cancellatum,* whilst *Montipora* is not the host. All the pieces of corals used in the settlement experiments were free of coral barnacles. Observations on the settlement of coral barnacles on their host and non-host corals can determine whether their settlement is specific to their preferred hosts. The settlement experiment was performed separately on the collected coral species cultured in natural filtered seawater at room temperature and pressure. The seawater was changed every 8 h. The number of cyprids settled on the corals after 24 h of exposure was recorded to determine the correlation between the coral species and cyprid settlement.

(e) Settlement and metamorphosis of barnacles on corals

To elucidate how the cyprids settle on their respective host coral, we introduced three-day-old cyprids of *N. grandis* and *P. cancellatum* to *Galaxea* and *Turbinaria* species hosts, respectively, to observe the settlement and metamorphosis process of barnacles. For each barnacle species, 10 individual cyprids were transferred into a small polycarbonate plastic container containing filtered seawater and a small piece of their respective host corals. The seawater was changed every 8 h. The settlement behaviours and metamorphic events of cyprids were recorded and photographed using a Leica M125 stereomicroscope (Leica, Germany) equipped with a custom-made C-mount and lens mount adapter attached to a Panasonic Lumix GH4 camera. All photomicrographs and video recordings were processed using Adobe Photoshop CS6, Adobe Illustrator CS6, and Corel VideoStudio Ultimate 2020.

(f) Growth of the vertical base length and shell width of juvenile barnacles

To infer the growth of the base and shell of coral-associated barnacles, the newly metamorphosed juveniles of *N. grandis* were removed from host corals using fine needles. The morphology of the base and shell of *N. grandis* was captured every two days using a stereomicroscope with an attached camera as described above. The vertical base length and shell width of juvenile barnacles were measured under digital photo with a scale bar, for growth pattern analyses. All statistical analyses on the growth of the base length and shell width of *N. grandis* were conducted in R^53^ using the package ggplot2^54^.

## Supplementary Information

Supplementary Information.

## Data Availability

All supplementary movies (Movie 1 to 5) are available on the Figshare repository at https://figshare.com/s/e9cccf4e596a29afbcf8.
