# Supplementary material for: Host specificity and adaptive evolution in settlement behaviour of coral-associated barnacle larvae (Cirripedia: Pyrgomatidae)
Source: Sci Rep. 2023 Jun 14;13:9668. doi: 10.1038/s41598-023-33738-3 (PMC10267106; doi:10.1038/s41598-023-33738-3)
Supplement: Supplementary file 1 — Supplementary Information. [file 41598_2023_33738_MOESM1_ESM.pdf]

Supplementary videos:

All supplementary movies (Movie 1 to 5) are available on the Figshare repository at https://figshare.com/s/e9cccf4e596a29afbcf8.

Movie Legend

Movie 1. Exploratory behavior of *Nobia grandis* cyprid on its coral host, *Galaxea* sp.

Movie 2. Inspection behavior and permanent settlement of *Nobia grandis* on its coral host, *Galaxea* sp.

Movie 3. Exploratory behaviour of *Pyrgoma cancellatum* on its coral host, *Turbinaria* sp.

Movie 4. Metamorphosis of settled cyprids of *Nobia grandis* on its coral host.

Movie 5. Permanent settlement and metamorphosis of *Pyrgoma cancellatum* cyprid.

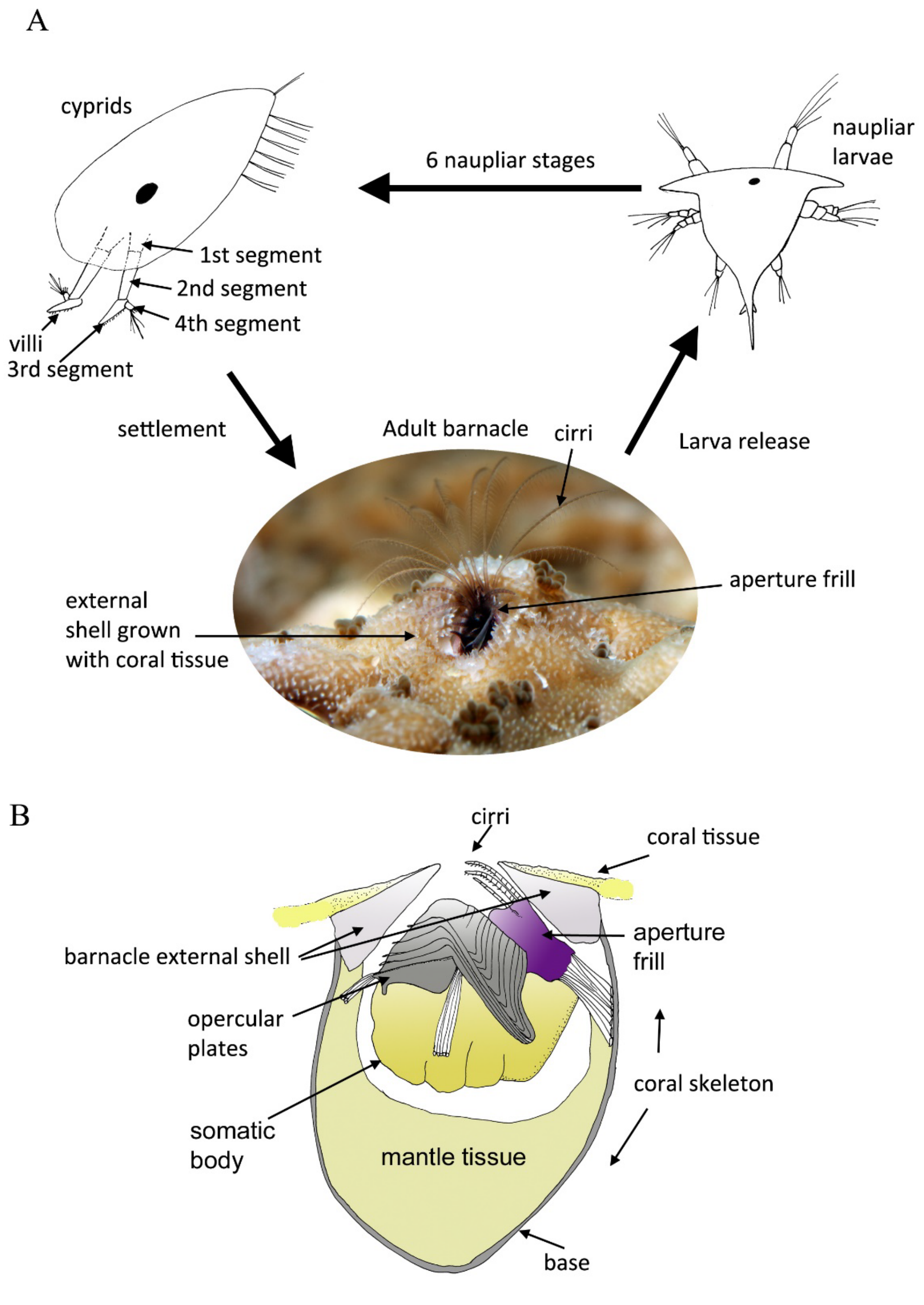

Figure S1 – A. Life cycle of pyrgomatid coral barnacles. Cyprids have a pair of antennules, which are consists of 4 segments. B. Longitudinal section of a pyrgomatid barnacle showing the base and other morphological parts.
